# Using Wearable Inertial Sensors to Estimate Kinematic Parameters and Variability in the Table Tennis Topspin Forehand Stroke

**DOI:** 10.1155/2020/8413948

**Published:** 2020-05-10

**Authors:** Ziemowit Bańkosz, Sławomir Winiarski

**Affiliations:** ^1^Department of Sports Didactics, Faculty of Sports, University School of Physical Education in Wrocław, Wrocław, Poland; ^2^Division of Biomechanics, Faculty of Physical Education, University School of Physical Education in Wrocław, Wrocław, Poland

## Abstract

The study examined kinematic parameters and their inter- and intrasubject variability in the topspin forehand of seven top-level table tennis players. A wireless inertial measurement unit (IMU) system measured the movement of the playing hand to analyze the Ready position, Backswing, and Forward events, and a racket-mounted piezoelectric sensor captured the racket-ball Contact. In a four-phase cycle (Backswing, Hitting, Followthrough, and Back to Ready position), body sensors recorded the cycle and phase duration; angles in the sagittal plane at the shoulder, elbow, and wrist of the playing hand and at the knee joints; and acceleration of the playing hand at the moment of racket-ball contact. The coefficient of variation (CV) was calculated to determine the variability of kinematic parameters within and between players. The observed variability in stroke time duration was low (CV < 20%) indicating constancy. The small-to-medium intraindividual variability of angles (CV < 40%) indicates that each player used a broadly repeatable technique. The large intraindividual variability in movement was probably functional (i.e., motor adjustment and injury avoidance). Interindividual and intraindividual variability of knee and elbow angles was low; wrist extension was the most variable parameter (CV > 40%) for all tasks, and shoulder joint variability was medium-to-large. Variability in hand acceleration was low (CV < 20%). Individual players achieved relatively constant hand acceleration at the moment of contact, possibly because angular changes at one joint (e.g., shoulder) could be compensated for by changes at another (e.g., wrist). These findings can help to guide the teaching-learning process and to individualize the training process.

## 1. Introduction

Table tennis is a very fast, varied, and complex game, requiring an immediate response to changing stimuli. The difficulty of the game is increased by the high speed and variety of ball rotation [1, 2]. Multiple factors affect performance in this sporting discipline, including the level of technical preparation, tactical thinking, motor skills, mental preparation, and physiological determinants [3]. At an elite level, competition (match) outcomes are often determined by very small differences and sometimes by moments of excellent performance, and many table tennis coaches and professionals have identified comprehensive and perfect technique as a prerequisite for high-level success [4, 5]. In general, technique is thought to determine tactical potential and likelihood of achieving champion status [6].

There is evidence that the topspin forehand is among the most frequently used strokes in modern table tennis, in both the first attack and its continuation or counter-attack [7–9]. In this stroke, the velocity of the racket at the moment of contact with the ball reaches 20 m/s; following impact, the ball reaches a velocity of up to 45 m/s, rotating at up to 140 revolutions per second [1, 10, 11]. Theoreticians and practitioners regard the topspin forehand as a complex stroke, involving a kinematic chain of proximal-to-distal sequences or a stretch-shortening cycle. The speed at which the racket hits the ball is primarily influenced by hip joint and body rotation, flexion and adduction at the shoulder joint, and flexion at the elbow joint [12, 13]. During a game, the player must react to different situations and associated changes in ball parameters such as speed, rotation, flight trajectory, point of contact with the table, and height of rebound. In deciding on the type of stroke, the player adjusts their movements, the angle of the racket, the force applied, and the direction of racket movement. For example, a player attacking with topspin against a backspin shot and hitting the ball below the line (surface) of the table must “open” the racket, hitting the ball close to its central line and directing the movement from the bottom upward. In contrast, when returning a topspin ball flying above the net line, they must close the racket, hitting the upper part of the ball and directing movement strongly forward. Deciding on the type of stroke may also involve other changes—for example, from a rotational to a direct hit—resulting in further alteration of motion parameters.

This complexity means that players must choose from a range of options while maintaining high movement accuracy. It is therefore interesting to explore variations in table tennis players' movements and the limits of this variation. Within the rich literature on movement variation, some researchers have approached this as a problem of movement “noise”—that is, as nontargeted variability resulting from a complex multijoint movement [14]. However, it is increasingly suggested that this variability (both inter- and intraindividual) may be a functional and purposeful response to different situations and requirements of the task, such as parameters of the flying ball or avoiding injury [14]. Others have emphasized the need for consistency and repeatability; for example, Whiteside et al. suggested that a consistent projection angle during service is critical for successful tennis performance [15]. Small differences in movement parameters may also indicate a compensation mechanism, as for example when a change in the range of motion at one joint is compensated by a change at another [16–20]. According to some researchers, human movement variability facilitates motor learning through active nervous system regulation [21, 22]. Functional variability of movement is also thought to change and develop with player age and experience [23]. There is also evidence that variability decreases when movement is accompanied by increased mental focus on a particular aspect of activity [24].

As well as works investigating the kinematics of table tennis strokes [10, 12, 25], a number of studies on stroke kinematics have examined the relationships between movement and work done or force generated, between force and racket speed, and between the kinetics of the upper limbs and other body segments [13, 26, 27]. To the best of our knowledge, however, the issue of movement variability in table tennis kinematics has not yet been intensively explored. Among existing studies, Bootsma and van Wieringen [28] referred to movement variability in the accuracy and time of movement of five table tennis players during a drive stroke (which can be described for present purposes as “light topspin”). They found that when forced to play accurately—that is, to hit a specified target—the spatial and temporal accuracy of players' movement was reduced in attempting to hit the target. At the same time, variability at the moment of contact between racket and ball was also reduced—a phenomenon they characterized as “compensatory variability.” In a study of racket kinematics and direction during the forehand drive stroke across different levels of expertise, Shepard and Lee also found that movement variation was reduced at the time of racket-ball contact [29]. They described this phenomenon as “funneling” and again noted the speed-accuracy trade-off.

It seems, then, that the mechanisms of movement variability in table tennis warrant more detailed investigation. In particular, it seems interesting to investigate the best table tennis players' use of the topspin forehand, which is the most commonly used stroke in the game. To guide the teaching-learning process and to individualize the training process, it seems useful to explore movement variability and the conditions and limits of its occurrence. This may assist in the process of monitoring and correcting technique and in developing improvement plans for individual players.

To that end, the present study employed inertial measurement unit (IMU) sensors from the myoMotion System to measure selected kinematic parameters of the topspin forehand stroke and the intra- and interindividual variability of these parameters among advanced male table tennis players. Specifically, we hypothesized that measurement of key kinematic parameters of the topspin forehand stroke (duration of the cycle and its phases and knee, shoulder, elbow, and wrist joint angles) would explain any variability in these strokes. We further assumed that the values of some of these parameters would vary more (CV > 40%)—especially in the Ready position and Backswing phases—and that some would be less variable (CV < 20%), especially the moment of contact and elbow and wrist joint angles, in light of the principle of “funneling” described in the literature.

## 2. Materials and Methods

**Figure 1:**
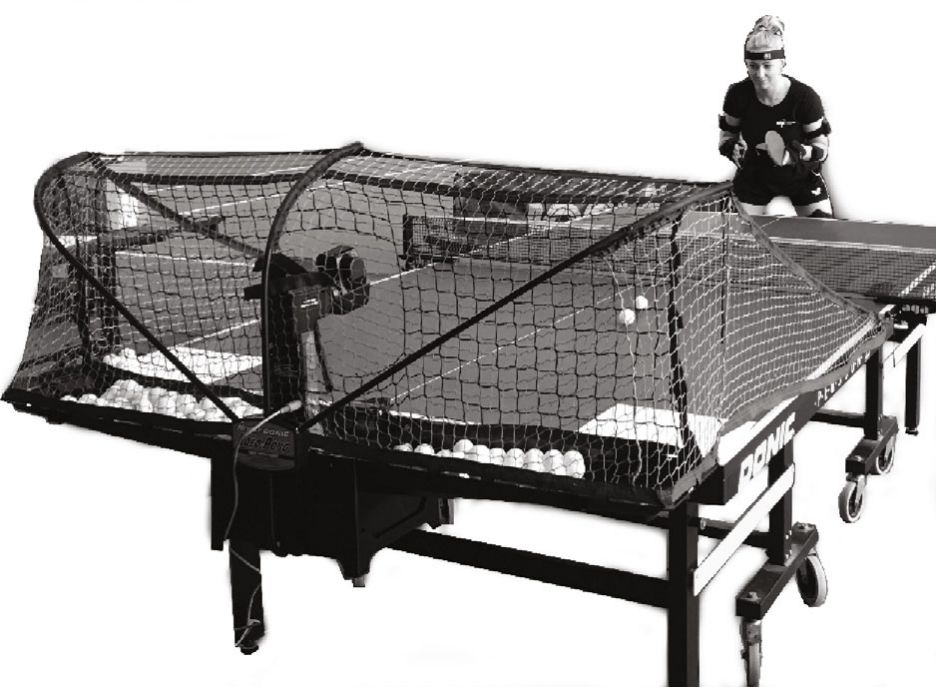
Research stand.

**Figure 2:**
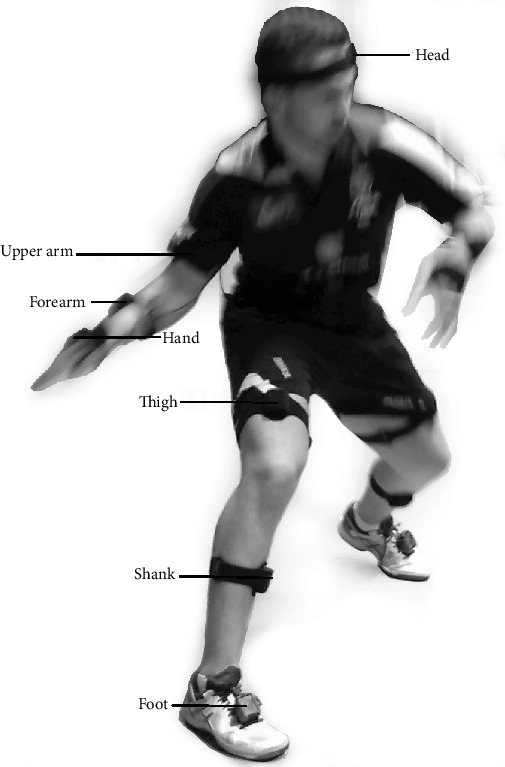
Sensor locations.

The study participants were seven top adult male players from Poland's national team, with a mean body height of 177 ± 3.5 cm and mean body mass of 76 ± 8.5 kg. Each participant was informed about the purpose and nature of the research and signed an informed consent form. The study protocol was approved by the Institutional Ethics Board (Senate's Research Bioethics Commission at the University School of Physical Education in Wrocław). All the players ranked among the top ten Polish senior athletes. Six of the players were right-handed, and one was left-handed. Participants were asked to perform the topspin forehand stroke with submaximal or maximal force on a specially prepared stand (see Figure 1), and individual kinematic parameters of the players were measured using the MR3 myoMuscle Master Edition system (myoMOTION™, Noraxon, USA). To record acceleration, wireless IMU sensors were attached (as per the myoMotion protocol described in the manual) to the following body segments: head, left and right arms, left and right forearms, left and right hands, left and right thighs, left and right foot, shanks, and body trunk (see Figure 2). The myoMotion system includes a set of 1 to 16 inertial sensors; using so-called fusion algorithms, a 3D accelerometer, gyroscope, and magnetometer measure the 3D rotation of each sensor in absolute space in terms of yaw, pitch, and roll (also known as orientation or navigation angles). To record and analyze the moment of racket-ball contact, a piezoelectric sensor (7BB-20-6L0, Murata Manufacturing Co., Ltd., USA) compatible with the myoMotion system was attached to the racket. The max sampling rate was 100 Hz per sensor for the whole 16-sensor set, and this was adjusted to the speed of registration by the piezoelectric sensor (1500 Hz). The maximum test range of the 3-axis digital accelerometer is ±16*g* (*g* = 9.8 m/s^2^) with 10000*g* high shock survivability.

Prior to testing, the athletes completed the standardized general (15 minutes) and sport-specific (20 minutes) warm-up procedures. Each then performed a topspin forehand with maximum or submaximal force. Each task comprised 15 presented strokes, and the player was required to hit the marked area (30 × 30 cm) at the corner of the table. Every successful shot (i.e., “on table” and played diagonally) was recorded for further analysis. Any balls missed, hit out of bounds, or hit into the net were excluded. Balls were delivered according to specified parameters (see Table 1) by a dedicated table tennis robot (Newgy Robo Pong Robot 2050, Newgy Industries, Tennessee, USA; see Figure 1).

All movement parameters were recorded and calculated using a standard protocol and report of the myoMotion software. Focusing on the topspin forehand technique, assessment of variability was confined to joints on the playing side (shoulder, elbow, and wrist) and the knee joints, which have been identified as decisive for performance of the topspin forehand [12, 30, 31]. We chose to discuss only selected movements in sagittal plane where the ROM is greatest and the speed of movement has probably the greatest impact on the spin of ball. In order to show the magnitude of variation, we chose only selected parameters. The sensors attached to the athlete's body and to the racket recorded the values of the following parameters for further analysis: angles of playing hand, extension of the wrist, shoulder flexion, elbow flexion, and knee flexion (both sides), and acceleration of the playing hand at the moment of racket-ball contact. Movement of the playing hand was measured to assess the following specific events in the cycle: Ready position (racket not moving after previous stroke, before swing, forward-backward acceleration =0); Backswing (the moment at which the racket changes direction from backward to forward in the sagittal plane following the swing); and Forward (the moment at which the racket changes direction from forward to backward in the sagittal plane after the stroke). The fourth event in the cycle—the moment of ball-racket contact—was captured by the racket-mounted sensor. Each click on the racket (i.e., contact of racket and ball) transmitted a signal from the sensor to the system software. The moment at which this signal was registered was treated as the moment of racket-ball contact.

By capturing these events, it was possible to determine the duration of individual phases of the stroke: Backswing (Ph1); Hitting (Ph2); Followthrough (Ph3); and Back to Ready position (Ph4). It is also worth noting that the study confirms the utility of Noraxon's IMU as an alternative to optical motion capture systems for movement analysis. During dynamic trials, the root mean square error (RMSE) for myoMotion (as compared to Vicon) was 0.50 deg, with a correlation coefficient of 0.99 between Vicon and myoMotion for dynamic trials [32].

Using basic descriptive statistics (means, standard deviations, and variances) for all kinematic parameters, their variability was measured as coefficients of variation [33]. For the purposes of this study, low variability was defined as CV < 20; medium variability was defined as 20–40; and high variability was defined as CV > 40. Statistical calculations were performed using the Statistica software (Statistica 12.5, StatSoft Inc., Tulsa, USA).

## 3. Results and Discussion

Intraindividual and interindividual variability in the topspin forehand stroke was measured by coefficients of variation (CV), based on IMU values for the following kinematic parameters.

### 3.1. Time Duration

The results for temporal parameters are shown in Tables 2 and 3.

There was little variation in overall cycle duration across participants (Table 2). Of the four distinct phases, the Hitting phase (Ph2) was shortest in duration. Variability in the duration of individual hitting phases was small (CV < 20%) or medium (20–40%). Values in Ph4 (return to the Ready position) differed for every player and returned the most cases of CV > 40%. Among individual players, variability in duration of the entire cycle and its individual phases (Table 3) was small (total time TT), with CV values for all players ranging from 0.8% to 6.7% (Table 3). Low variability cases included Ph1 (one player), Ph2 (four players), and Ph3 (six players). The remaining cases in these three phases were characterized by medium variability. Based on these results, the large number of cases of low variability (low CV values) in individual athletes for the entire duration of the stroke (TT) and for most phases (mainly Hitting and Followthrough) indicates that variation in these parameters is small and that stroke characteristics are fairly constant, confirming the findings of previous studies [11, 13]. For each player, the greatest variation was observed in duration of Ph4 (Back to Ready position). The beginning of the Ready position phase (Ph4) was defined as the point at which the player held the racket stationary before the next action (forward − back acceleration = 0) while waiting for the robot to deliver the ball. As this moment was freely determined by each participant, the duration of this phase varied more. Interestingly, the results across the entire group indicate small or medium variation in duration for most phases (Table 3) other than Ph4 (from Forward to Ready position), where variability exceeded 40%. This indicates that players' performance of the tasks was similar in terms of duration of the stroke and its individual phases.

### 3.2. Angles

The myoMotion system was also used to measure angles at joints known to be important for specific events during table tennis performance (see Tables 3 and 4). In the analysis of results for the entire group (intervariability), knee and elbow joints accounted for the highest number of cases of small variability (low CV value) (see Table 4). There were 8 cases of high or very high variability and 12 cases of small or average variability. In terms of intraindividual variability, the analysis indicates that individual variability of movement was low in 82 of 140 cases and medium in 19 cases (Table 5). Regarding individual events, there were some cases of high variability for all joints, most of which related to angles in the Ready position (6 of 35 cases) and at the moment of contact (14 of 35 cases) (see Table 4). High variability most often related to the position of the hand at the wrist joint on adopting the Ready position (2 of 7 cases), completion of the movement (Forward, 6 of 7 cases), and the position of the arm at the shoulder joint at the moment of Backswing (3 of 7 cases).

The analysis of angle variations in the four selected topspin forehand events (Ready position, Backswing, Contact, and Forward) focused on the CV values of the angles. Intraindividual variability was more often small or medium rather than large, indicating that the participating players each used a repeatable technique. As in other sports, however, it is impossible to state unequivocally that any given player repeated the same task with the same movement pattern. For example, in their review of research on interindividual and intraindividual variation in track and field throwing events, basketball throws, and gait during human locomotion, Bartlett et al. demonstrated that the large variation in movement is probably functional in character, as athletes make motor adjustments or seek to avoid injury [14]. They also noted that even the best athletes (with similar results) fail to perfectly reproduce the same movement (in terms of parameters, range of motion, and coordination). Bartlett et al. further argued that these factors should be considered when preparing an individualized training plan for each athlete, taking into account their unique capabilities. In the present context, that might include addressing the various ways of coordinating topspin movement and perhaps compensating for a small range of motion in one joint by ensuring a larger range of motion in another. Crucially, any coaching to shape and improve stroke technique should be flexible.

### 3.3. Acceleration and Compensatory Mechanism

The variability of acceleration values was small in all cases, both for the entire group and for individual players (Table 6). It is important to mention that the specified task required participants to use submaximal force. At the moment of contact, several players exhibited high or very high variability of angles, especially in extension at the wrist joint. There was also medium and high variability of the shoulder joint in many cases, but the variability of acceleration values remained low, perhaps because changes at the shoulder and wrist joints are mutually dependent—in other words, changes at one joint are compensated for by changes at the other. This kind of compensation mechanism has been observed in other studies and in other sports; for example, Button, MacLeod, Sanders, and Coleman evaluated movement variability in basketball players performing free throws [34] and found that players compensated for mutual changes of angle at the elbow and wrist joints. They further reported that variability at the elbow and wrist joints tended to increase toward the end of the throwing action. In a study of cueing actions in billiards (assessing parameters such as velocity, acceleration, height, and angle of the cue), Kornfeind et al. [35] observed significant variability in stroke movement despite very similar outcome values.

Many researchers have emphasized functional variability—that is, flexible changes in movement parameters in response to the changing requirements of the game or competition [14, 19, 36]. In the present case, the observed acceleration values may indicate similar functional variability and compensation mechanisms in table tennis. While angular variability at the joints was often low or medium in individual athletes, the frequency of high variability cases indicates that table tennis players' technique is not entirely repetitive. In contrast, there was very little difference in hand acceleration at the time of contact, with CV values well below 10%. Despite some angular variation in subsequent events, individual players (and the entire group) exhibited relatively constant hand acceleration at the moment of contact between racket and ball, indicating compensatory changes in angular parameters (e.g., shoulder/wrist) as observed in many other sports [16–19, 37, 38].

In sporting contexts, there is some evidence of the need for constancy and repeatability in the range of specific parameters [15]; in the present case, one such constant element was acceleration value at the moment of contact, with small CV values across the entire group. A similar phenomenon has been documented in billiards [36], golf [20], basketball [31], and by other authors [14]. The low CV values for acceleration at so important a point as racket-ball contact support the findings of Bootsma and Wieringen [29] and Shepard and Lee and Xie [30] regarding acceleration and reduced variability at critical moments.

Among the limitations of the present study, the sample was small (*n* = 7), and all of the participants were male, making it difficult to generalize the findings. Additionally, while this study examined only the topspin forehand with use of submaximum or maximum force, our recent work reports similar findings for other variants of this stroke [39]. A final limitation is that the present study was laboratory-based, and examination of variability in kinematic parameters under game condition might yield different outcomes.

## 4. Conclusions

In this study of the table tennis topspin forehand, the use of an IMU system facilitated measurement of the duration of individual phases and key kinematic parameters, as well as estimation of their variability. The low CV values for duration of most phases (mainly Hitting and Followthrough) for both individual athletes and the entire group indicates small variability in this constant stroke characteristic.

Intraindividual variability of angles was most often low or medium, indicating repeatable technique among the participating players. Nevertheless, it is impossible to state unequivocally that any player repeated the same task with the same movement pattern. As the literature suggests, the large variability in movement may be functional and compensatory in character, reflecting motor adjustment of various parameters.

Inter- and intraindividual variability of joint angles was generally low for the knees and the elbow joint. The greatest observed variability was in extension at the wrist joint, with medium or large variability of the shoulder joint in many cases. It seems likely that the observed changes at the shoulder and wrist joints are mutually dependent (i.e., changes at one joint are compensated for by changes at the other).

There was low variability in hand acceleration. Despite the variability of some angles in subsequent events, it can be concluded that individual players achieved relatively constant hand acceleration at the moment of contact between racket and ball. This indicates compensatory changes in angular parameters at one joint to offset changes at another.

## Figures and Tables

**Table 1:** Table tennis robot parameters.

Robot parameter	Value
Rotation (direction of spin)	Topspin
Speed (determines both speed and spin, where 0 is the minimum and 30 is the maximum)	18
Left position (left most position to which the ball is delivered)	4
Wing (robot's head angle indicator)	8.5
Frequency (time interval between balls thrown)	1.4

**Table 2:** Time duration of particular phases during topspin forehand in the entire group of players (*n* = 7)—means, standard deviations (SD), variations (V), and coefficients of variation (CV).

Variable	Topspin forehand				
	Ph1	Ph2	Ph3	Ph4	TT
Mean (s)	0.5	0.1	0.2	0.4	1.5
SD (s)	0.1	0.0	0.0	0.1	0.0
V	0.0	0.0	0.0	0.0	0.0
CV (%)	18.3^∗∗^	46.2	18.2^∗∗^	25.7^∗^	1.4^∗∗^

Ph1: Backswing; Ph2: Hitting; Ph3: Followthrough; Ph4: Back to Ready position; TT: total time of the cycle. ^∗^Average variability. ^∗∗^Small variability. Not marked CV: high and very high variability.

**Table 3:** Variability (CV in %) of time duration of particular phases during topspin forehand in particular players (1-7).

Player	Topspin forehand				
	Ph1	Ph2	Ph3	Ph4	TT
1	31.6^∗∗^	10.0^∗∗^	21.4^∗^	107.8	2.3^∗∗^
2	22.1^∗^	36.8^∗^	3.1^∗∗^	65.9	1.1^∗∗^
3	25.3^∗^	36.0^∗^	1.6^∗∗^	79.9	5.0^∗∗^
4	21.6^∗^	13.8^∗∗^	5.2^∗∗^	64.4	0.8^∗∗^
5	15.9^∗∗^	15.5^∗∗^	2.8^∗∗^	63.7	0.9^∗∗^
6	22.2^∗^	30.8^∗^	8.9^∗∗^	65.2	6.3^∗∗^
7	28.4^∗^	9.2^∗∗^	6.7^∗∗^	80.1	6.7^∗∗^

Ph1: Backswing; Ph2: Hitting; Ph3: Followthrough; Ph4; Back to Ready position; TT: total time of the cycle; ^∗^Average variability. ^∗∗^Small variability. Not marked CV: high and very high variability.

**Table 4:** Values of angles at joints in chosen events during topspin forehand in the entire group of players (*n* = 7)—means, standard deviations (SD), variations (V), and coefficients of variation (CV).

	Ready position					Backswing					Contact					Forward				
	ShF	ElF	WrE	RKnF	LKnF	ShF	ElF	WrE	RKnF	LKnF	ShF	ElF	WrE	RKnF	LKnF	ShF	ElF	WrE	RKnF	LKnF
Mean (deg)	13.2	66.1	44.7	43.3	41.4	8.7	47.6	25.4	51.9	58.0	26.4	43.7	47.7	47.6	52.7	90.8	87.0	-3.0	51.1	49.7
SD (deg)	9.2	6.6	41.5	10.6	5.3	8.6	20.9	11.8	14.3	8.9	11.8	15.3	39.3	12.7	9.3	18.2	21.5	25.2	10.8	10.1
V	84.3	43.9	1727.8	111.5	28.1	74.9	435.0	139.3	203.6	78.7	139.1	232.8	1542.5	162.4	87.3	330.9	464.1	636.2	117.2	102.7
CV (%)	69.6	10.0^∗∗^	92.4	24.4^∗^	12.8^∗∗^	99.2	43.8	46.5	27.5^∗^	15.3^∗∗^	44.6	34.9^∗^	82.3	26.8^∗^	17.7^∗∗^	20.0^∗^	24.7^∗^	851.0	21.2^∗^	20.4^∗^

ShF: shoulder flexion; ElF: elbow flexion; WrE: wrist extension; RKnF: right knee flexion; LKnF: left knee flexion. ^∗^Average variability. ^∗∗^Small variability. Not marked CV: high and very high variability.

**Table 5:** Values of angles at joints in chosen events during topspin forehand of particular players (1-7)—means, standard deviations (SD), variations (V), and coefficients of variation (CV).

Variable		Ready position					Backswing					Contact					Forward				
		ShF	ElF	WrE	RKnF	LKnF	ShF	ElF	WrE	RKnF	LKnF	ShF	ElF	WrE	RKnF	LKnF	ShF	ElF	WrE	RKnF	LKnF
1	Mean (deg)	10.8	62.2	82.2	44.7	42.1	36.1	75.8	6.0	74.8	65.8	34.9	56.7	88.5	71.7	59.6	82.1	108.5	40.2	66.9	57.6
	SD (deg)	11.6	11.9	33.9	16.6	13.2	32.7	6.2	43.2	3.4	6.9	15.8	26.1	39.9	30.8	27.6	24.2	19.6	74.3	10.9	9.3
	V	135.1	141.2	1148.4	276.5	173.6	1067.2	38.6	1862.9	11.3	48.3	251.1	681.1	1593.4	947.4	759.6	586.3	383.7	5521.9	118.9	86.5
	CV (%)	147.7	19.0^∗∗^	49.1	33.4^∗^	28.1^∗^	138.3	8.4^∗∗^	185.9	4.5^∗∗^	10.7^∗∗^	55.7	55.4	54.8	54.3	55.3	32.3^∗^	18.4^∗∗^	308.3	16.9^∗∗^	16.5^∗∗^

2	Mean (deg)	18.5	80.3	40.0	59.5	40.2	0.6	58.1	31.6	70.6	71.6	17.2	61.0	62.1	64.3	70.4	70.2	72.2	-8.4	50.5	61.8
	SD (deg)	19.6	3.6	10.1	3.8	7.3	30.5	19.2	23.7	7.2	6.0	22.6	21.5	16.0	2.7	4.1	36.7	2.7	11.5	4.7	5.2
	V	386.0	12.6	103.0	14.2	53.1	930.5	370.3	563.8	52.0	35.6	509.8	463.6	255.5	7.3	16.7	1344.6	7.4	132.5	21.9	27.0
	CV (%)	144.6	4.4^∗∗^	23.1^∗^	6.4^∗∗^	17.7^∗∗^	931.5	35.04	88.0	10.4^∗∗^	8.4^∗∗^	181.5	41.5	24.1^∗^	4.2^∗∗^	5.8^∗^	48.5	3.8^∗∗^	131.3	9.0^∗∗^	8.4^∗∗^

3	Mean (deg)	17.2	68.8	33.9	38.1	50.0	11.5	67.5	40.4	47.9	57.7	-1.4	43.9	5.9	42.4	61.3	75.9	104.5	11.9	58.6	47.8
	SD (deg)	3.5	7.6	8.1	4.5	4.7	2.4	2.4	8.3	3.6	5.1	3.4	22.2	4.4	21.2	29.5	2.3	8.9	6.6	5.7	7.7
	V	12.2	57.1	65.5	20.0	22.2	5.6	5.7	68.9	12.6	26.4	11.6	493.9	19.6	450.0	871.6	5.2	78.9	43.9	32.6	58.5
	CV (%)	20.8^∗^	11.1^∗∗^	24.2^∗^	11.3^∗∗^	9.3^∗^	19.2^∗∗^	3.5^∗∗^	19.1^∗∗^	7.6^∗∗^	8.9^∗∗^	147.0	66.4	78.0	66.6	65.7	3.0^∗∗^	8.3^∗∗^	48.5	9.8^∗∗^	15.7^∗∗^

4	Mean (deg)	14.6	67.3	-1.5	48.1	36.1	12.8	13.9	23.4	48.6	50.0	19.1	18.6	24.2	45.6	43.0	87.8	60.9	12.1	52.2	37.0
	SD (deg)	6.0	17.7	2.7	10.0	6.4	7.6	7.0	3.0	16.7	6.7	6.0	7.2	6.8	17.3	12.9	3.9	3.5	6.1	16.8	4.9
	V	35.4	311.6	7.1	100.6	40.4	57.8	49.4	8.9	277.9	45.2	36.4	52.3	45.7	297.8	165.7	15.2	12.0	37.6	281.6	23.9
	CV (%)	41.8	27.8^∗^	214.5	22.5^∗^	16.6^∗∗^	59.3	47.9	13.0^∗∗^	37.8^∗^	13.4^∗∗^	32.7^∗^	40.5	30.4^∗^	44.9	31.0^∗^	4.4^∗∗^	5.7^∗∗^	52.6	36.8^∗^	12.7^∗∗^

5	Mean (deg)	21.1	61.8	79.5	46.1	40.4	6.0	48.3	30.3	49.8	55.2	10.3	62.3	54.9	49.5	50.5	107.1	113.8	-48.3	56.6	57.5
	SD (deg)	3.5	4.9	6.3	3.9	7.6	3.8	6.2	6.3	3.2	5.1	4.6	4.4	4.8	3.1	6.5	2.4	4.4	7.7	4.3	4.7
	V	12.0	23.6	40.1	15.6	57.8	14.5	38.8	40.3	10.3	25.8	21.2	19.7	23.1	9.7	42.8	5.9	19.0	59.6	18.2	22.6
	CV (%)	16.9^∗∗^	7.9^∗∗^	7.9^∗∗^	8.5^∗∗^	20.0^∗∗^	60.7	13.1^∗∗^	21.1^∗^	6.5^∗∗^	9.2^∗∗^	46.1	7.1^∗∗^	8.8^∗∗^	6.3^∗∗^	12.7	2.3^∗^	3.8^∗∗^	15.2^∗∗^	7.5^∗∗^	8.18^∗∗^

6	Mean (deg)	0.8	60.6	102.7	39.3	38.7	22.3	29.0	26.8	45.5	44.1	27.2	35.3	88.9	48.9	52.9	135.2	71.8	-27.7	52.2	49.7
	SD (deg)	2.6	3.4	8.7	5.0	3.5	2.9	4.0	3.5	3.1	3.7	2.5	5.2	6.2	5.1	3.4	37.6	4.7	20.2	6.4	4.6
	V	6.5	11.8	75.4	25.4	12.2	8.2	16.4	12.1	9.8	13.7	6.3	26.9	38.3	25.8	11.6	1412.7	22.5	408.1	41.4	21.3
	CV (%)	356.6	5.6^∗∗^	8.7^∗∗^	13.1^∗∗^	9.3^∗∗^	12.4^∗∗^	13.8^∗∗^	12.6^∗∗^	7.0^∗∗^	8.2^∗∗^	9.1^∗∗^	14.1^∗∗^	7.0^∗∗^	10.6^∗∗^	6.3^∗∗^	30.7^∗^	6.6^∗∗^	83.0	12.6^∗∗^	9.3^∗∗^

7	Mean (deg)	22.1	73.8	-6.5	26.7	33.2	-14.2	34.7	11.2	36.3	66.6	2.6	38.0	11.6	35.2	65.9	94.7	88.0	12.8	29.6	36.0
	SD (deg)	9.7	4.6	5.2	6.2	4.6	4.6	5.6	5.8	1.1	3.1	3.4	5.9	4.8	1.4	2.6	4.6	5.0	6.9	3.4	3.7
	V	94.4	20.8	27.5	38.4	20.7	21.3	30.9	33.2	1.2	9.8	11.4	34.3	23.2	1.8	6.6	21.0	25.3	47.5	11.4	14.0
	CV (%)	43.7	6.7^∗∗^	76.3	22.9^∗^	13.8^∗∗^	35.1^∗^	15.1^∗∗^	48.4	3.0^∗∗^	4.7^∗^	121.2	14.7^∗∗^	42.0	3.9^∗∗^	3.9^∗∗^	4.8^∗∗^	5.8^∗∗^	57.3	11.3^∗∗^	10.4^∗∗^

ShF: shoulder flexion; ElF: elbow flexion; WrE: wrist extension; RKnF: right knee flexion; LKnF: left knee flexion; ^∗^Average variability; ∗∗Small variability; not marked CV: high and very high variability.

**Table 6:** Values of acceleration of “playing hand” in the moment of racquet's contact with the ball—entire group and particular players—means, standard deviations (SD), variations (V), and coefficients of variation (CV).

	Variable	Topspin forehand
Entire group (*n* = 7)	Mean (m/s^2^)	149.2
	SD (m/s^2^)	8.6
	V	73.7
	CV (%)	5.8^∗∗^

Players		
1	Mean (m/s^2^)	159.4
	SD (m/s^2^)	3.1
	V	9.6
	CV (%)	2.0^∗∗^

2	Mean (m/s^2^)	160.1
	SD (m/s^2^)	14.3
	V	204.6
	CV (%)	9.2^∗∗^

3	Mean (m/s^2^)	158.9
	SD (m/s^2^)	3.6
	V	12.9
	CV (%)	2.3^∗∗^

4	Mean (m/s^2^)	156.0
	SD (m/s^2^)	6.8
	V	46.0
	CV (%)	4.3^∗∗^

5	Mean (m/s^2^)	138.4
	SD (m/s^2^)	10.1
	V	101.5
	CV (%)	7.3^∗∗^

6	Mean (m/s^2^)	157.9
	SD (m/s^2^)	1.8
	V	3.1
	CV (%)	1.1^∗∗^

7	Mean (m/s^2^)	148.3
	SD (m/s^2^)	14.3
	V	204.7
	CV (%)	9.6^∗∗^

^∗^Average variability. ^∗∗^Small variability. Not marked CV: high and very high variability.

## Data Availability

The raw data.xls data used to support the study findings are included in the supplementary information file (available here).
